# Living human lung slices for ex vivo modelling of lung cancer

**DOI:** 10.1172/jci.insight.190703

**Published:** 2025-07-29

**Authors:** Siavash Mansouri, Annika Karger, Clemens Ruppert, Marc A. Schneider, Andreas Weigert, Rajender Nandigama, Blerina Aliraj, Lisa Strotmann, Anoop V. Cherian, Diethard Pruefer, Peter Dorfmuller, Ludger Fink, Ibrahim Alkoudmani, Stefan Gattenlöhner, Bastian Eul, Andre Althoff, Peter Kleine, Hauke Winter, Andreas Guenther, Hossein-Ardeschir Ghofrani, Soni S. Pullamsetti, Friedrich Grimminger, Werner Seeger, Rajkumar Savai

**Affiliations:** 1 Institute for Lung Health (ILH), Justus Liebig University, Giessen, Germany.; 2 Max Planck Institute for Heart and Lung Research, Member of the German Center for Lung Research (DZL), Member of the Cardio-Pulmonary Institute (CPI), Bad Nauheim, Germany.; 3 Department of Internal Medicine, Member of the DZL, Member of CPI, Justus Liebig University, Giessen, Germany.; 4 Translational Research Unit, Thoraxklinik at Heidelberg University Center, Heidelberg, Germany.; 5 Goethe University Frankfurt, Faculty of Medicine, Institute of Biochemistry I, Frankfurt, Germany.; 6 Department for Immunity of Inflammation, Mannheim Institute for Innate Immunoscience (MI3), Medical Faculty Mannheim, Heidelberg University, Mannheim, Germany.; 7 Translational Lung Research Center (TLRC) Heidelberg, German Center for Lung Research (DZL), Department of Thoracic Surgery, Thoraxklinik at the University Hospital Heidelberg, Heidelberg, Germany.; 8 Division of Systems Biology of Signal Transduction, German Cancer Research Center (DKFZ), Heidelberg, Germany.; 9 Department of Thoracic Surgery, Kerckhoff Heart and Thorax Center, Bad Nauheim, Germany.; 10 Institute of Pathology, Dermatopathology, Cytology and Molecular Pathology, UEGP, Wetzlar, Germany.; 11 Department of General and Thoracic Surgery, University Hospital Giessen, Giessen, Germany.; 12 Department of Pathology, Justus Liebig University, Giessen, Germany.; 13 Department of Pulmonology, Thoraxzentrum Offenbach, and; 14 Department of Thoracic Surgery, Thoraxzentrum Offenbach, Sana Klinikum Offenbach, Offenbach, Germany.; 15 Department of Thoracic Surgery, Thoraxklinik at Heidelberg University Hospital, Heidelberg, Germany.; 16 Pulmonary Hypertension and Vascular Biology Research Group of Quebec Heart and Lung Institute, Department of Medicine, Laval University, Quebec, Canada.

**Keywords:** Immunology, Oncology, Immunotherapy, Lung cancer

## Abstract

The tumor microenvironment (TME) markedly affects cancer progression, yet traditional animal models do not fully recapitulate the situation in humans. To address this, we developed tumor-derived precision lung slices (TD-PCLS), an ex vivo platform for studying the lung TME and evaluating therapies. TD-PCLS, viable for 8–10 days, preserve the heterogeneity and metabolic activity of primary tumors, as confirmed by seahorse analysis. Using multispectral FACS and phenocycler multiplex imaging, we spatially profiled TME components and cancer cell functionality. Additionally, TD-PCLS revealed patient-specific responses to chemo- and immunotherapies. To complement TD-PCLS, we established tumor-cell–seeded PCLS (TCS-PCLS) by introducing tumor and immune cells into healthy lung slices. This model highlighted macrophage-tumor interactions as critical for tumor cell proliferation, migration, and immune modulation. Together, these platforms provide a robust tool for lung cancer research, enabling precision medicine and advancing therapeutic discovery.

## Abstract 2

We developed patient-derived precision lung slices, preserving architecture and heterogeneity, enabling in situ studies of patient cells and therapies for advanced immunotherapeutic evaluation.

## Graphical abstract

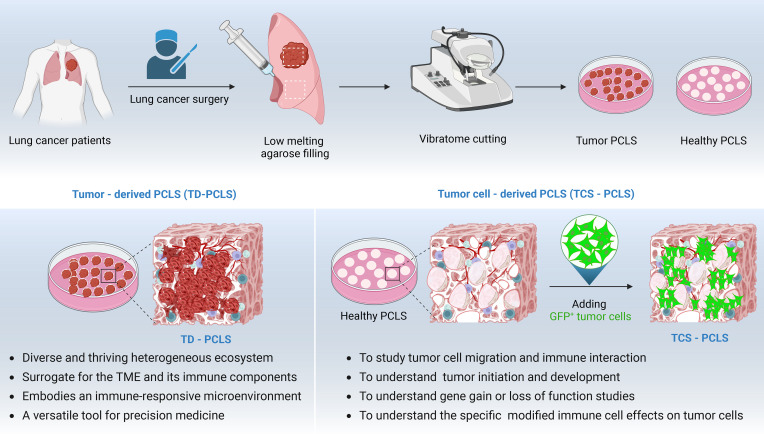

## Introduction

Lung cancer has the highest tumor-associated mortality rate worldwide, though in the last 2 decades, the mortality rate of lung cancer patients decreased due to the therapeutic shift from traditional cytotoxic chemotherapy to immunologic and targeted therapies (1). This change in approach is based on 2 new insights into lung tumors, firstly the identification of lung tumor–driving mutations in protooncogenes and tumor suppressor genes, and secondly, the recognition that, in addition to neoplastic cancer cells, other cellular entities, particularly immune and stromal cells as part of the tumor microenvironment (TME), have a significant impact on the growth and metastasis of lung tumors (2). Targeted therapies focusing on specific gene mutations, together with immunotherapies targeting components of the TME, have shown promising results in the treatment of lung cancer. However, only a subgroup of patients responds fully to these therapies, particularly immunotherapies, suggesting that unknown aspects of the lung TME may interfere with treatment (3). More importantly, the unique characteristics of an individual’s TME are critical to treatment outcomes and may be considered a major determinant of treatment efficacy in each lung cancer patient.

The majority of recently developed cancer drugs fail in phase 2 and 3 clinical trials and do not receive clinical approval. Lung cancer in particular has one of the lowest approval rates from phase 1 trials compared with other cancers (4, 5). One of the major challenges in this area is the lack of model systems that accurately represent individual tumor characteristics, which is critical for predicting the efficacy of new therapeutic agents. This contributes to the high rate of unsuccessful phase 1 clinical trials in lung cancer.

Currently, animal models and 2D/3D cell culture techniques are the predominant systems for evaluating treatment responses in-vitro. However, significant discrepancies exist between human and mouse innate and adaptive immune responses (6), which are critical, given the role of immune cells in the lung TME and their impact on immunotherapy outcomes (3). Furthermore, despite the considerable genetic similarity at the sequence and functional level between humans and mice, differences in genomic regulation and interaction may occur. These differences could potentially impact outcomes in mouse models of lung cancer by differentially influencing the evolutionary development of genetic mutations in mice and humans (7). Such differences could lead to unique metabolic profiles in the human TME compared with the mouse TME, as cellular metabolism is closely linked to cellular and organism-wide immune states and genetic background. It is known that mice have generally a higher metabolic rate and develop obesity faster than humans (8). All of these factors contribute to the low success rate of only 8% in translating preclinical results from mice to humans in the field of lung cancer (9).

The disadvantages of 2D and 3D cell culture methods in the simulation of human lung tissue pose significant limitations in cancer research. In 2D cultures, primary human lung cells preserve certain morphological and functional features of lung tissue, but the absence of TME components, especially immune and stromal cells, and the lack of a spatial architecture that mimics 3D cellular interactions pose the greatest challenges (10). 3D models may thus have advantages over 2D systems, particularly when mimicking the spatial structures of the lung that allow cells to grow into multiple directions, promoting more physiologically relevant cellular interactions corresponding to the in vivo microenvironment of the lung. This increased structural complexity allows the cells to maintain a natural shape, particularly in relation to the lung airway tree, alveolar structure, and organization, resulting in greater similarity to the real system in vivo in terms of physiological function (11). Nevertheless, these models are not free from shortcomings, as they often fail to account for crucial elements such as the unique genetic background of individual patients and various cellular components of the lung TME, such as immune cells, stromal cells, and vascular cells. Despite the fact that 3D models represent an improvement over 2D cultures, they need further refinement to accurately replicate the complex cellular interactions and therapeutic responses in living lung tissue.

The recently developed ex vivo model known as precision-cut lung slices has advanced our understanding of molecular mechanisms and therapeutic approaches (12–17). PCLS are carefully prepared, thin, uniform slices of human or animal lung tissue that are now widely recognized. PCLS of the lung preserve the microenvironment of the original tissue, including the immune, endothelial and stromal cells, as well as the alveolar and distal airway structure and extracellular matrix (ECM). In addition, the interactions between different cellular components of the lung and the interactions with the ECM are preserved in the PCLS model (14). PCLS derived from human donors also capture the unique genetic background of individual patients, making them invaluable for both basic and translational research on lung diseases, as shown, for example, for chronic obstructive pulmonary disease (COPD), idiopathic pulmonary fibrosis (IPF) and pulmonary hypertension (13, 18). To date, however, a comprehensive methodological approach for the use of tumor-derived precision lung slices (TD-PCLS) and tumor-cell–seeded PCLS (TCS-PCLS) in lung cancer research has been lacking. To address this gap, we have developed a detailed step-by-step protocol for obtaining, culturing, and utilizing TD-PCLS and TCS-PCLS for basic and translational studies in lung cancer.

## Results

### Harvesting TD-PCLS from fresh human lung tumors.

**Figure 1:**
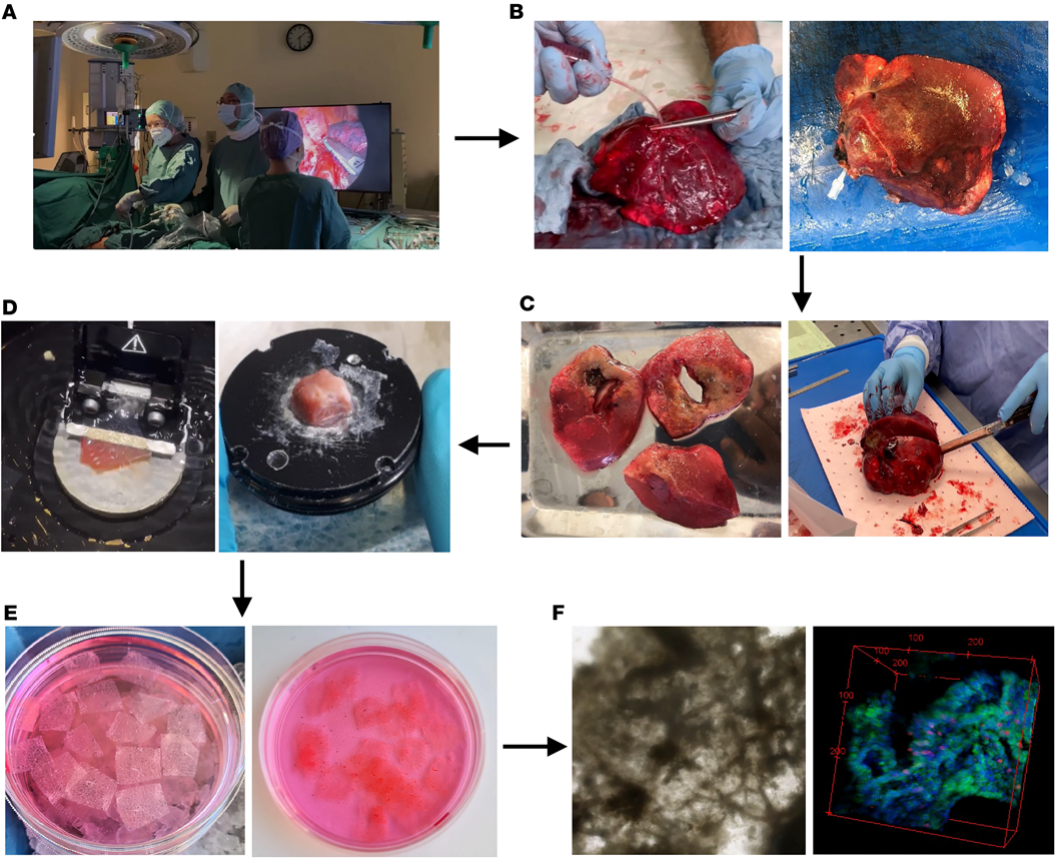
Preparation and culture of TD-PCLS from fresh human lung tumor. (**A** and **B**) Immediately after lobectomy, the removed lung was filled with 1% low-melting agar in the operating room or pathology department and waited for the agar to solidify. (**C**) The filled lung was evaluated and sectioned by the expert pathologist to identify the tumor area for clinical purposes and also to provide the proper piece for TD-PCLS preparation. (**D**) The tumor piece was transferred on ice to the molecular biology laboratory. First, the tumor was cut in the shape with sharp angle (about 90 degrees) and then stuck to the metal plate, which was then moved into the vibratome sample box filled by cold PBS. (**E** and **F**) TD-PCLS was prepared by cutting tumor pieces in various thickness, depending on tumor type and structure and subjected to further treatment, imaging, and analysis. The thickness was between 200–500 μm from lung adenocarcinoma. The criteria that defines the thickness was tumor size, thickness, solidity, and necrotic area.

Immediately after lung cancer surgery, the excised lung tissue was directly infused with 1% low-melting agarose and left to solidify by cooling down with ice (Figure 1, A and B). Once solidified, the lung tissue was examined by an expert pathologist to identify the tumor area, ensuring that a suitable sample was obtained both for clinical evaluation and for TD-PCLS preparation (Figure 1C). The identified tumor sample was then cut at a precise angle and affixed to a metal plate (Figure 1D). This assembly was placed in the vibratome sample box filled with cold PBS (Figure 1D), and then the tumor was sliced into TD-PCLS of various thicknesses (Figure 1E), depending on the tumor’s type and structure, cultured in conventional cell culture media and conditions (37°C and 5% CO_2_) and was used for further experimental procedures (Figure 1F).

### TD-PCLS is a dynamic and vital heterogeneous ecosystem.

**Figure 2:**
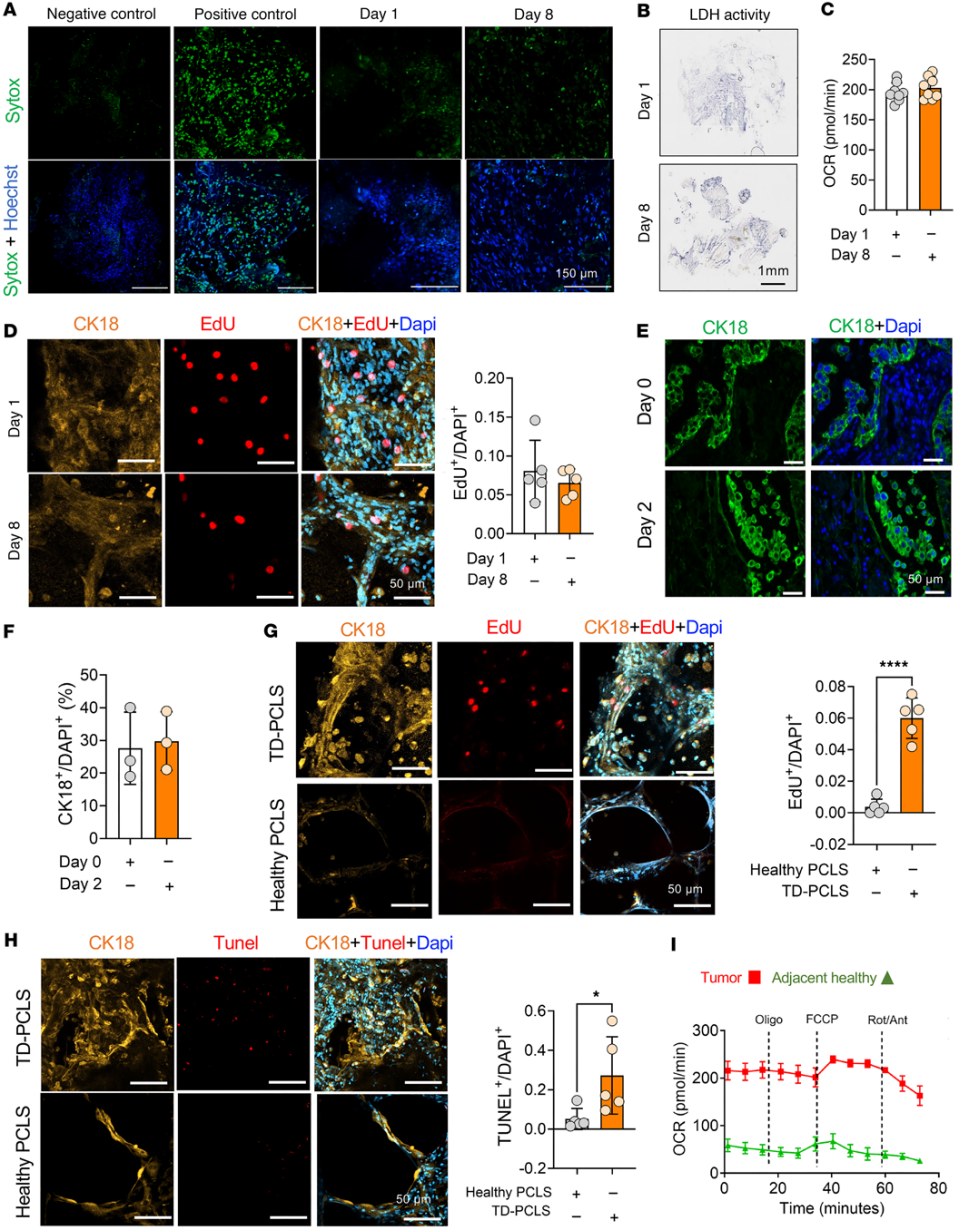
TD-PCLS is a dynamic and vital heterogeneous ecosystem. (**A**) Sytox (green) and Hoechst (blue) immunofluorescence staining as viability indicator in TD-PCLS during culture for 8 days. (*n* = 3 lung adenocarcinoma tumors). Scale bar: 150 μm. (**B**) In situ lactate dehydrogenase (LDH) activity in TD-PCLS during culture for 8 days. Scale bar: 1mm. (**C**) Oxygen consumption rate (OCR) in TD-PCLS cultured for 1 and 8 days measured via Seahorse analysis, (*n* = 2 lung adenocarcinoma patients). (**D**) Representative images (left panel) with quantification (right panel) of TD-PCLS cultured for 1 and 8 days, stained with EdU (red), cytokeratin 18 (CK18) and Dapi (blue). Scale bar: 50μm. *n* = 5 technical replicates. (**E** and **F**) Immunofluorescence staining and quantification of positive cytokeratin 18 (CK18, tumor cell marker; green) in formalin-fixed paraffin-embedded (FFPE) of lung tumor tissue immediately after surgery (day 0) and after processed to TD-PCLS and culture for 2 days. Dapi was used as nuclear dye (blue). Scale bar: 50 μm. (**G** and **H**) Immunofluorescence staining and quantification of EdU (proliferation marker; red), Tunel (apoptosis marker; red) in positive cytokeratin 18 (CK18, tumor cell and epithelial marker; orange) in TD-PCLS and healthy. Dapi was used as nuclear dye (blue) (*n* = 3 lung adenocarcinoma tumors). Scale bar: 50μm. (**I**) OCR measurement of tumor areas punched from TD-PCLS and healthy adjacent area punched from healthy PCLS (*n* = 3 lung adenocarcinoma tumors). Statistical significance was determined by a 2-tailed unpaired Student’s *t* test. Data are presented as mean ± SEM. *P < 0.05, ****P <0.0001.

To evaluate the viability of TD-PCLS as a model system, we monitored its viability during culture using Sytox immunofluorescence staining. The results showed that TD-PCLS can be maintained for 8–10 days under standard cell culture conditions, as evidenced by an increase in Sytox signals (Figure 2A). In addition, the activity of lactate dehydrogenase (LDH), a key enzyme of the glycolysis in TD-PCLS, was found to maintain stability over 8–10 days (Figure 2B). We also checked cellular metabolism through measuring oxygen consumption rate (OCR) level and cellular proliferation (positive for EdU and CK18; cytokeratin 18) after 8 days culture ex vivo. We did not find any significant changes about OCR level and proliferation rate between day 1 and day 8 of ex vivo culture (Figure 2, C and D). Furthermore, we also tested the integrity of tumor structure after slicing with the vibratome and culturing as TD-PCLS for 48 hours. Indeed, TD-PCLS maintain tumor structure in ex vivo culture environment, as shown by cytokeratin 18–positive (CK18-positive) cells, which are structurally present in the TD-PCLS as in the tumor tissue (Figure 2, E and F). We also checked the proliferative and apoptotic state in TD-PCLS compared with healthy PCLS. Our analysis focused on tumor cells contained in the TD-PCLS and epithelial cells in healthy PCLS, asking for the capability not only proliferate (positive for EdU and CK18; cytokeratin 18), but also to undergo apoptosis (positive Tunel and CK18). The data showed TD-PCLS and healthy PCLS not only contained proliferative cells but also apoptotic cells which both are enriched in TD-PCLS compared with healthy PCLS (Figure 2, G and H). In addition, cellular metabolism data documented OCR in the TD-PCLS, indicating functionality, especially in the tumor area, which has higher mitochondrial activity than adjacent healthy areas (Figure 2I). Interestingly, we detected metabolic heterogeneity in different tumor areas of the TD-PCLS (Supplemental Figure 1A; supplemental material available online with this article; https://doi.org/10.1172/jci.insight.190703DS1). Some regions exhibited high metabolic activity and clearly responded to mitochondrial stress; OCR levels decreased after inhibition by oligomycin and increased after treatment with FCCP, indicating active mitochondrial function, while other areas did not at all respond to FCCP (Supplemental Figure 1A). We also tested how the TD-PCLS thickness can impact metabolic and proliferation status within TD-PCLS. There were not any significant differences of OCR level between 200 μm to 500 μm thickness of TD-PCLS. Furthermore, proliferation rate also was similar between 250 μm and 500 μm (Supplemental Figure 1, B and C). Overall, these results highlight that TD-PCLS represent a viable and metabolically active model system, mimicking the spatial heterogeneity of a real lung tumor.

### TD-PCLS is a proxy for TME and immune constituents.

**Figure 3:**
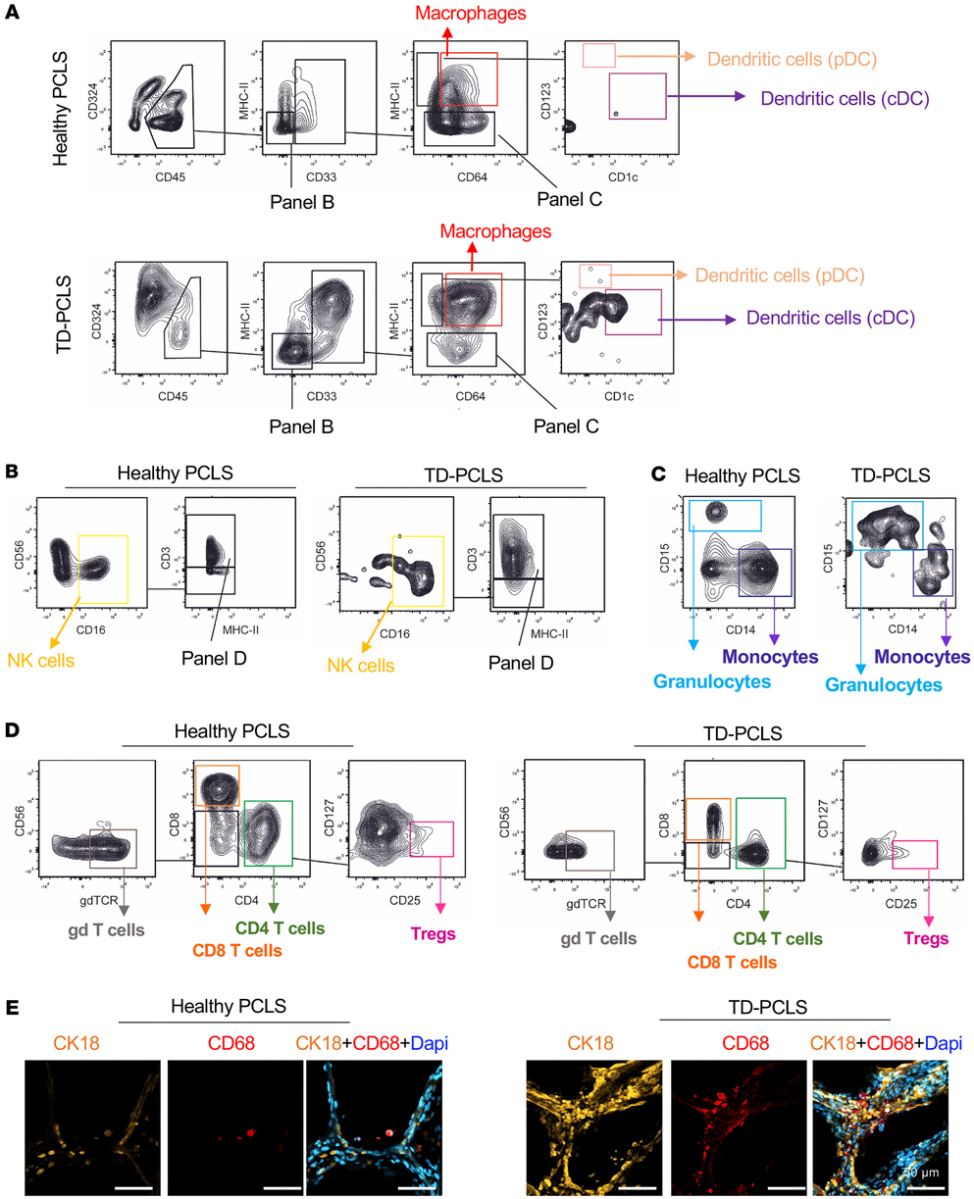
TD-PCLS is a proxy for TME and immune constituents. Representative plots of spectral flow cytometry of Healthy- and TD-PCLS showing the gating strategy of immune cell components including (**A**) macrophages, plasmacytoid dendritic cells (pDCs), classical dendritic cells (cDC), (**B**) natural killer (NK) cells, (**C**) monocytes, granulocytes, (**D**) gd-T cells, CD8^+^ T cells, CD4^+^ T cells and regulatory T cells (Tregs), after 2-days’ culture (*n* = 3 lung adenocarcinoma tumors). (**E**) Immunofluorescence staining of cytokeratin 18 (CK18, tumor cell marker; orange) and CD68 as macrophage pan marker (red) in healthy- TD-PCLS. Dapi was used as nuclear dye (blue) (*n* = 3 lung adenocarcinoma tumors). Scale bar: 50 μm.

Given the key role that immune cells within the TME structure of the lung play in therapy response, we tested whether TD-PCLS and healthy PCLS could maintain the immune profile of the lung’s TME during culture. We established a tissue dissociation method that allowed FACS analysis, showing that TD-PCLS and healthy PCLS contain most of the cellular components of the lung TME and overall microenvironment after 2 days of ex vivo culture, including macrophages, plasmacytoid dendritic cells (pDCs), classical dendritic cells (cDC), natural killer (NK) cells, monocytes, granulocytes (Figure 3, A–C), gd-T cells, CD8^+^ T cells, CD4^+^ T cells, and regulatory T cells (Tregs) (Figure 3D). In particular and similar to our previous findings (19, 20), we could find the strong difference of epithelial compartment (CD324^+^ cells, Figure 3A), myeloid (Figure 3, A–C), and lymphoid (Figure 3D) cell infiltration in TD-PCLS compared with healthy PCLS. To map the tissue distribution of immune cells, we also checked the macrophage population as the most dominant immune cells in lung TME by immunofluorescence (IF) imaging in TD-PCLS and healthy PCLS. We not only detected enriched macrophages as CD68^+^ cells in TD-PCLS compared with healthy PCLS (Figure 3E), but also documented the subpopulations of antitumor M1 macrophages (IL8) and tumor-promoting M2 macrophages (ALOX15 or CD206) in TD-PCLS (Supplemental Figure 2A), as to be expected for lung tumor tissue (21). We also could determine tumor cells (CK18) in TD-PCLS in the vicinity of different macrophage subpopulations (Supplemental Figure 2A), also known for in vivo lung cancer topography. Therefore, by using FACS analysis and IF imaging in TD-PCLS, we demonstrated that TD-PCLS preserves tumor cells as well as the major immune components of the lung TME.

### TD-PCLS represents an immune interactive microenvironment.

**Figure 4:**
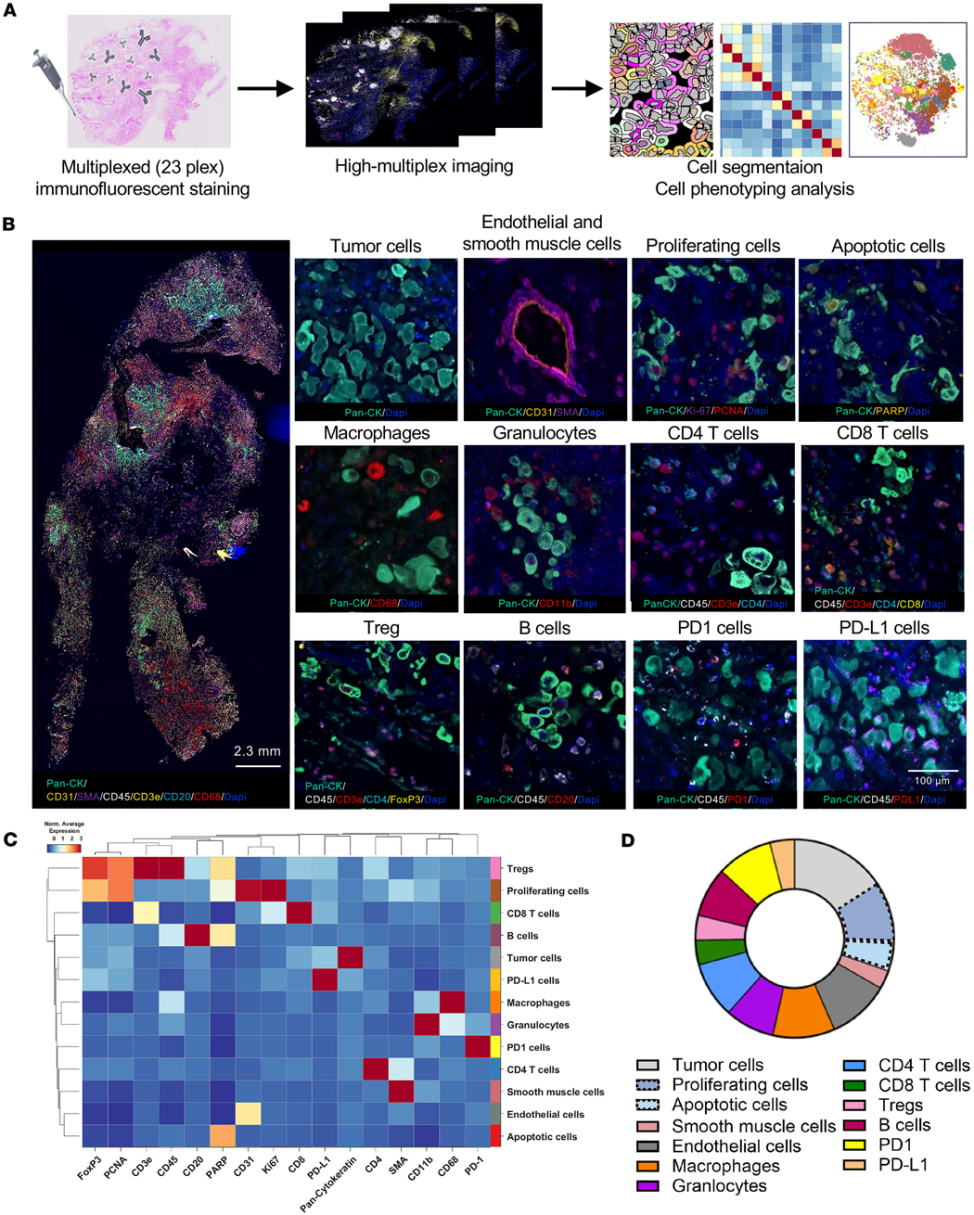
Multiplex IF and multispectral imaging show distribution of structural, functional, and immune cell phenotypes in TD-PCLS. (**A**) Workflow of multiplex IF staining in TD-PCLS with Akoya Bioscience technology. (**B**) Representative images of different cell phenotypes including tumor cells (Pan-CK), endothelial cells (CD31), smooth muscle cells (SMA), proliferating tumor cells (Pan-CK, PCNA and Ki67), apoptotic tumor cells (Pan-CK and PARP), granulocytes (CD11b), macrophages (CD68), CD4^+^ T cells (CD45, CD3e, and CD4), CD8^+^ T cells (CD45, CD3e, CD4, and CD8), Tregs (CD45, CD3e, CD4, and FoxP3), B cells (CD45 and CD20), PD1 (CD45 and PD1) cells, and PD-L1 cells (CD45 and PD-L1) in TD-PCLS. Scale bars: 2.3 mm for the whole tumor image (left) and 100 μm (right, individual cell type). (**C** and **D**) Heatmap shows a curated clustering dendrogram with cell phenotypes and pie chart showing the abundance of each cell phenotype in TD-PCLS tissue.

Next, we asked whether TD-PCLS can preserve the cellular spatial resolution. For that, we applied HiPLEX immunophenotyping by Phenocycler on TD-PCLS (Figure 4A). To this end, we stained TD-PCLS with 16 antibodies after 2 days of culture (Figure 4A). The panel covers the various structural and immune cell components. Accordingly, we could detect the tumor cells (Pan-CK; pan-cytokeratin), endothelial cells (CD31), and smooth muscle cells (SMA; smooth muscle actin) in TD-PCLS (Figure 4, B and C). We could group the tumor cells phenotypically to proliferating cells (Pan-CK, PCNA; proliferating cell nuclear antigen and Ki67) and apoptotic cells (Pan-CK and PARP; poly ADP ribose polymerase). Regarding immune cells, TD-PCLS was enriched with different type of immune cells including macrophages (CD68), B cells (CD45 and CD20), and granulocytes (CD11b) (Figure 4, B and C). We also detected T cell subpopulations, including CD4^+^ T cells (CD45, CD3e, and CD4), CD8^+^ T cells (CD45, CD3e, CD4, and CD8), and Tregs (CD45, CD3e, CD4, and FoxP3). Interestingly, TD-PCLS also preserve PD1 cells (CD45 and PD1) and PD-L1 cells (CD45 and PD-L1) as 2 main cell populations involved in immunotherapy regimens (Figure 4, B and C). In addition, the spatial architectural analysis revealed the organized structure of lung TME with distinct proportion of immune cell infiltration, tumor cell clusters, and structural cells. Among immune cells, macrophages and CD4^+^ T cells were the most prominent immune cells, 10% and 9% of all the cell types, respectively (Figure 4D). The high abundance of structural cells including endothelial cells, around 10%, represents the preserved structural components in TD-PCLS. Finally, tumor cells represent the highest abundant cells, around 13% of all cell types. Interestingly, we could detect around 12% proliferating cells, which shows the viability and dynamics of TD-PCLS (Figure 4D). Altogether, spatial cellular phenotyping demonstrated that TD-PCLS maintain the complex and interactive lung TME not only with respect to immune cells, but also regarding proliferating tumor cells and structural cells. In addition, tPLCS has the potential to preserve subpopulations of T cells as a master regulator of immunotherapy.

### TD-PCLS is a toolbox for precision medicine.

**Figure 5:**
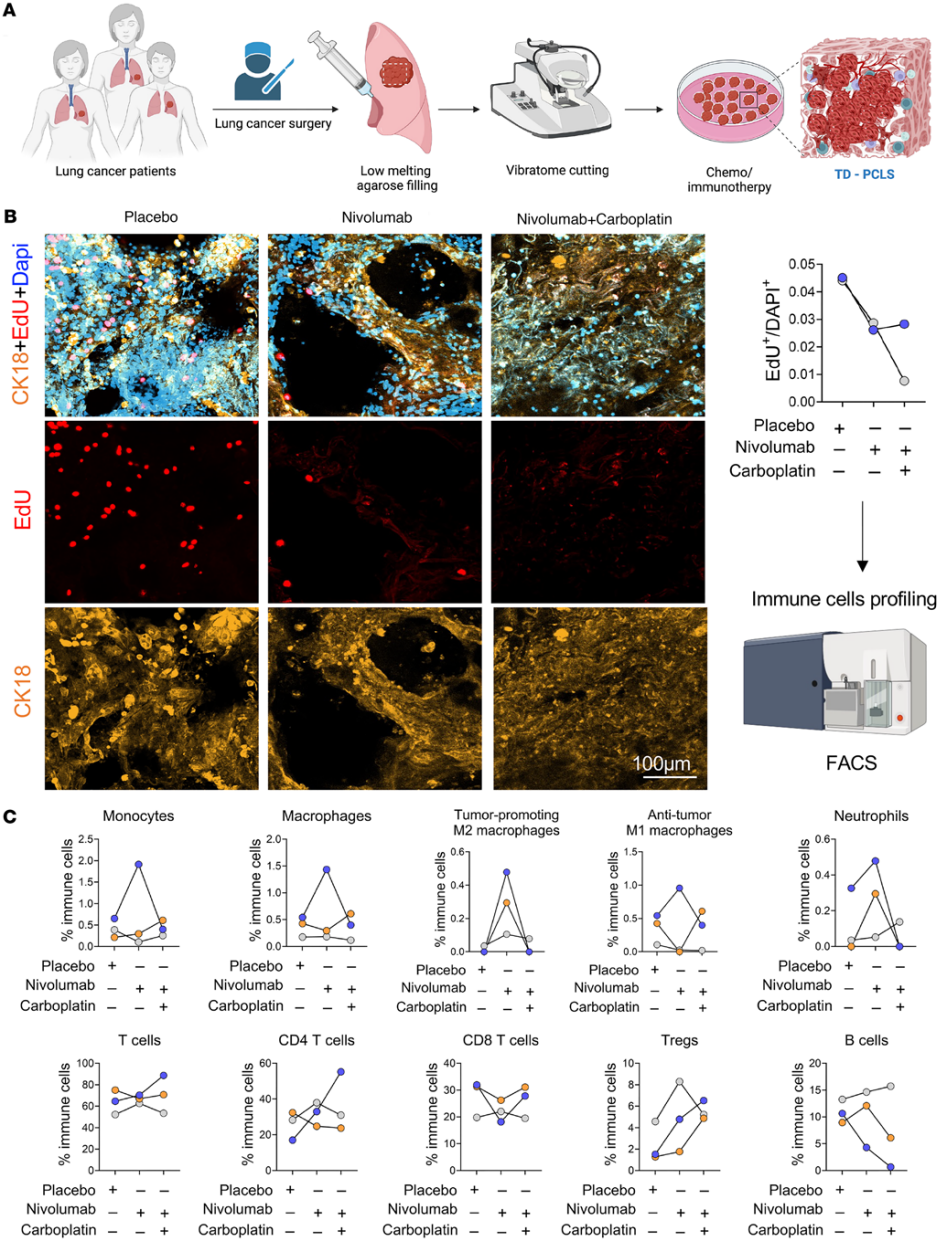
Treatment of TD-PCLS with chemo/immunotherapy regimens. (**A**) Workflow of chemo/immune therapy in TD-PCLS. Created in Biorender.com. (**B**) EdU staining (red) as marker of proliferating tumor cells stained with CK18 (orange) as tumor cell marker in TD-PCLS treated with nivolumab (10 μg/mL) and combination of nivolumab (10 μg/mL) +carboplatin (5 μg/mL) for 48 hours (*n* = 2 lung adenocarcinoma tumors) followed by quantification analysis. In the quantification (right panel), each color (grey or blue) indicate one patient. Dapi was used as nuclear dye (blue). Scale bar: 100 μm. (**C**) Immune profile analysis including monocytes, macrophages, tumor-promoting M2 macrophages, antitumor M1 macrophages, neutrophils, T cells, CD4^+^ T cells, CD8^+^ T cells, Tregs, B cells of TD-PCLSs treated with nivolumab (10ug/mL) and combination of nivolumab (10 μg/mL) +carboplatin (5 μg/mL) for 48 hours subjected to the established tissue dissociation protocol followed by spectral flow cytometry analysis (*n* = 3 lung adenocarcinoma tumors).

To assess whether immune cellular components in TD-PCLS are functional and responsive to lung cancer therapies, we treated TD-PCLS derived from 3 lung cancer patients with nivolumab, a PD-1 immune checkpoint inhibitor, and a combination of nivolumab and carboplatin, a chemotherapy agent, for 48 hours (Figure 5A). The effects of these treatments on tumor cell proliferation and immune cell populations within the TD-PCLS were analyzed using our established FACS method (Figure 5A). With regard to tumor cell proliferation under the 2 treatment strategies, this was reduced by nivolumab, as obvious from reduction of EdU staining in positive CK18 tumor cells in both patients, while combination therapy reduced the proliferation rate of tumor cells in only one patient (Figure 5B).

Regarding macrophages and monocytes, mostly patient 3 (marked in blue in Figure 5C) responded to the nivolumab therapy by increasing the number of macrophages and monocytes, and carboplatin masked the nivolumab effect by reducing the number of monocytes and macrophages in that patient. Interestingly, nivolumab elevated tumor-promoting M2 macrophage numbers in all patients, while the combination therapy returned them to levels similar to the untreated group in patient 3 and patient 2 (marked in orange in Figure 5C). Responses of antitumor M1 macrophages to the treatment strategies were more variable; in patient 3, nivolumab increased antitumor M1 macrophages, with the combination therapy reducing them to control levels. In patient 2, nivolumab decreased antitumor M1 macrophages, and the combination therapy slightly elevated them above control levels (Figure 5C). However, the number of antitumor M1 macrophages in patient 1 (marked with grey) was very low compared to other patients (Figure 5C). Regarding neutrophils, nivolumab increased the neutrophil populations in patients 2 and 3, and the combination therapy further enhanced this effect in patient 1, while it reduced neutrophil levels to the level of the untreated group in patients 2 and 3 (Figure 5C).

Concerning the T cell population, nivolumab and combination therapy did not alter the total number of T cells, with only patient 3 showing a slight increase in T cell numbers with the combination therapy. However, T cell subpopulations were affected by both nivolumab and the combination therapy. Notably, nivolumab enhanced the CD4^+^ T cell levels in patients 1 and 3, with no effect in patient 2, whereas the combination therapy led to a further increase in CD4^+^ T cells in patient 3. Regarding CD8^+^ T cells, nivolumab was effective in patients 2 and 3, reducing CD8^+^ T cell numbers, while the combination therapy increased CD8^+^ T cell numbers again to similar levels as the untreated group (Figure 5C). Notably, nivolumab increased the Treg population in all 3 patients, while the combination therapy reduced Treg numbers in patient 1 and increased them in patients 2 and 3 (Figure 5C).

Lastly, nivolumab increased the B cell numbers in patient 2, while the combination therapy reduced these numbers below control levels (Figure 5C). However, in patient 3, B cell numbers were reduced in both treatment groups: nivolumab and combination therapy (Figure 5C). Overall, the analysis of the 3 lung cancer patients across different treatment groups indicated that combination therapy was most effective in patient 3, who also showed a stronger response to nivolumab compared with the other 2 patients. Indeed, this patient has a higher PD-L1 level based on a histopathology report, without any reported mutation (Table 1). However, due to the limited number of patient samples in our study, further research is required to more robustly characterize the observed heterogeneity in response and its correlation with patient- and tumor-specific characteristics.

Together, these FACS immune analyses underscore the potential of using TD-PCLS as a model for individualized evaluation of the efficacy of immunotherapeutic strategies in lung cancer, particularly by demonstrating the heterogeneous response among patients, one of the main challenges in clinical trials and lung cancer treatment. The overall results indicate that immune cells in TD-PCLS are functional and can respond to immunotherapy (nivolumab) and combination therapy (nivolumab + carboplatin). Furthermore, our findings highlight the response heterogeneity to treatment approaches among TD-PCLS from each patient.

### Preparing TCS-PCLS from healthy PCLS.

**Figure 6:**
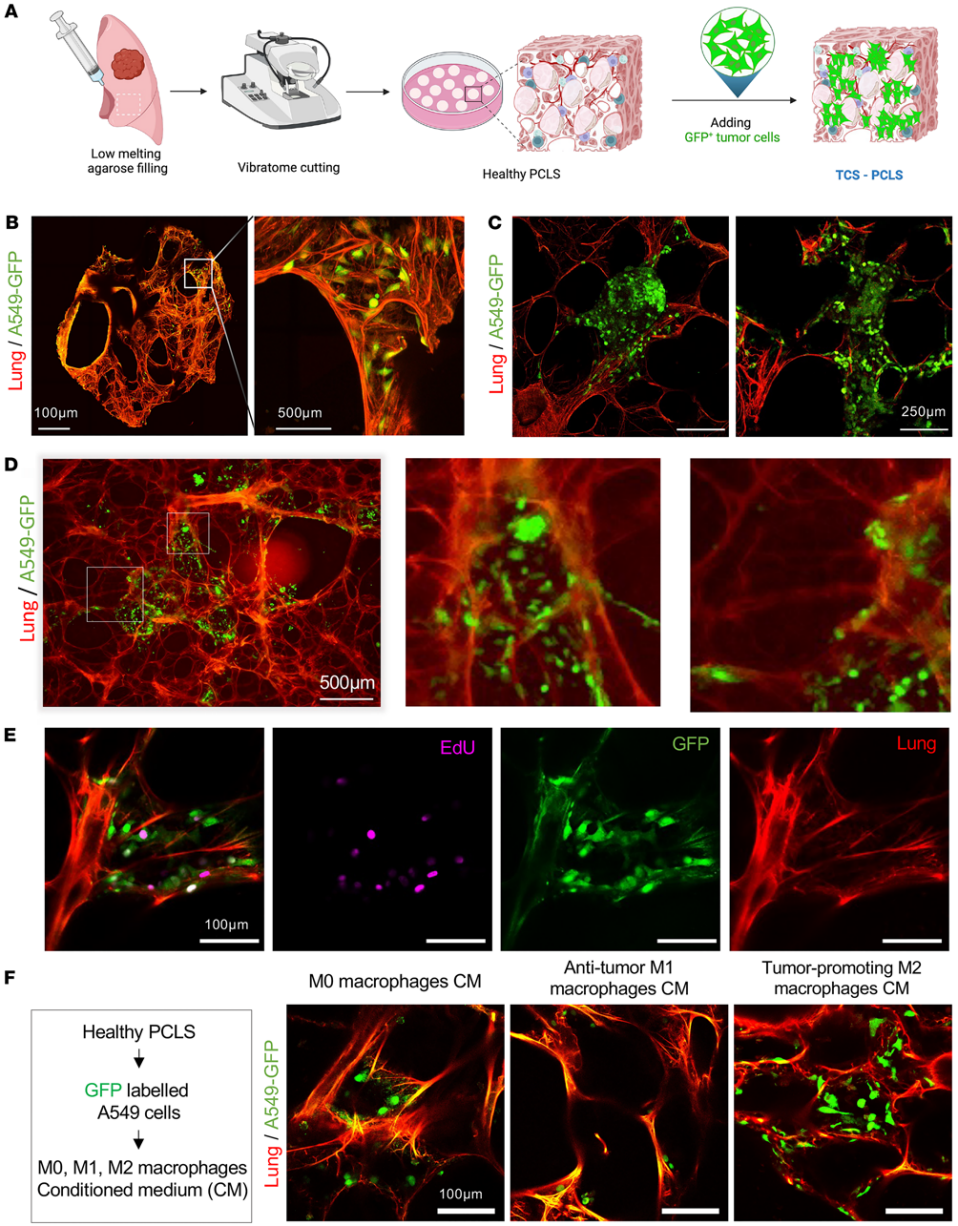
Preparation and applications of TCS-PCLS. (**A**) Workflow of preparation of TCS-PCLS by implementation of A549-positive GFP on nontumor/healthy PCLS. Created in Biorender.com. (**B**) Healthy PCLS was seeded with A549-GFP cells. After 24 hours, tissue was fixed and imaged with the Leica Thunder Imager using the lung autofluorescence (red), and the GFP signal emitted from A549 cells (green). Scale bar: 500 μm (right), 100 μm (left, magnification). (**C**) Healthy PCLS was cultured with A549-GFP spheroids for 24 hours. Spheroid outgrowth was imaged with the Leica SP8 confocal microscope using the Lung autofluorescence (red) and the GFP signal (green). Scale bar: 250 μm. (**D**) Healthy PCLS was seeded with A549-GFP cells. Time lapse imaging was done with the Keyence New All-in-One Fluorescence Microscope BZ-X800 in a stage top incubator for 24 hours. Scale bar: 500μm. (**E**) Healthy PCLS was seeded with A549-GFP cells and cultured for 24 hours. Prior fixation, 20 μM EdU was added overnight and visualized using the Click-iT EdU Kit. Slices were imaged using the Leica SP8 confocal microscope for the Lung autofluorescence (red), GFP signal (green), and EdU incorporation (magenta). Scale bar: 100 μm. (**F**) Healthy PCLS was seeded with A549-GFP and then cultured in different types of macrophage-conditioned medium (CM) for 24 hours, including naive unstimulated M0 macrophages, tumor-promoting M2 macrophages (stimulated with IL4 for 24 hours), antitumor M1 macrophages (stimulated with LPS and IFN-**γ** for 24 hours). Slices were imaged using the Leica SP8 confocal microscope. Scale bar: 100 μm.

To develop a human lung cancer ex vivo model system, we utilized PCLS derived from lung tissue not infiltrated by or adjacent to the tumor regions of lung cancer patients, combined with a green fluorescence-tagged lung cancer cell line (A549-GFP). Based on our protocol, we added A549-GFP cancer cells on top of the PCLS and allowed them to infiltrate and integrate into the lung structure (Figure 6A). We could successfully culture and image lung cancer cells within the human 3D PCLS tissue matrix (Figure 6B). The A549-GFP cells efficiently infiltrated and distributed throughout the lung structure of PCLS (Figure 6B). This model system offers significant advantages for assessing various tumor cell functions, including cell migration using tumor cell spheroids (Figure 6C). Imaging of tumor cell spheroids one day after being applied within the PCLS showed that they started to develop and expand (Figure 6C). Furthermore, time lapse imaging of PCLS (Supplemental Video 1), which are subjected to A549-GFP inoculation, revealed the migration of cancer cells within PCLS during culture (Figure 6D). Next, we also evaluated whether the embedded A549-GFP are proliferative. Indeed, EdU staining showed that A549-GFP reserved their proliferative potential after being added to PCLS (Figure 6E). Consequently, this allows for high-throughput readouts of treated engineered TD-PCLS, to be conducted efficiently and in a time-saving manner.

### Exposure of TCS-PCLS to macrophage supernatants.

Treatment of TCS-PCLS with conditioned media (CM) from different macrophage phenotypes demonstrated impact on the number of lung tumor cells attaching and growing in the lung tissue (Figure 6F). Notably, CM derived from tumor-promoting human M2 macrophages (stimulated with IL4 for 24 hours) resulted in the highest number of cancer cells (A549-GFP) in the tissue while CM derived from antitumor human M1 macrophages (stimulated with LPS and IFN-**γ** for 24 hours) resulted in a smaller number of cancer cells, both compared with TCS-PCLS treated with CM derived from naive human M0 macrophages (Figure 6F). Altogether, these findings highlight the utility of this model system in investigating the impact of various immune cell phenotypes on tumor growth in a real lung environment. This provides the advantage of maintaining a complex cellular composition, enabling comprehensive studies on tumor-immune interactions.

## Discussion

The advent of TD-PCLS as an ex vivo model for lung cancer research signifies a crucial step forward in bridging the gap between preclinical findings and clinical applications. This study delineates the comprehensive methodological framework and the promising utility of TD-PCLS in mirroring the complex lung TME, highlighting that (a) TD-PCLS maintain cellular viability and metabolic activity for up to 8–10 days under standard cell culture conditions; (b) TD-PCLS preserve the dynamic and patient-specific metabolic profile of lung tumors, found to show substantial individual variation; (c) TD-PCLS retain the diverse cellular components and interactions as well as their spatial architecture within the lung TME, including various immune cell populations; (d) TD-PCLS represent a model for evaluating immunotherapeutic strategies, which was demonstrated through treatments with nivolumab and a combination of nivolumab and carboplatin, as well as exposure to immune cell supernatants; and (e) TCS-PCLS represent an additional valuable tool, allowing “on-line” imaging and profiling of tumor development by inoculating engineered cancer cells in a natural lung microenvironmental architecture.

Interactions between tumors and their surrounding stroma are central to the development, spread, and resistance to treatment of many solid tumors, often resulting in therapeutic failure (22). Understanding these interactions is crucial for developing therapies that could counteract the tumor-promoting effects of the microenvironment. Biologically relevant 2D and 3D in vitro models (tumor spheroids, patient-derived tumor organoids, and cancer-on-a-chip) are central to this effort (23). Advances in polymeric scaffolds, 3D bioprinting, and organ-on-a-chip technologies have significantly improved in vitro modelling, especially when incorporating patient-derived cells to better simulate tumor–stroma dynamics (24). Overall, recent technological progress in bioengineered 3D models provides an enhanced platform for studying cancer biology, revealing key molecular pathways and offering new avenues for targeted treatment, although various questions remain open. In spheroid-based models, different cell types can be cocultured to form multicellular heterotypic spheroids, but with respect to the lung the cellular and structural features, may not fully mimic the complex architectural microenvironment of this complex organ. Organoid models are typically derived from epithelial progenitor cells and therefore lack an immune-competent microenvironment and stromal components (25). This limitation can be addressed by introducing stromal cells through 2D coculture techniques (26, 27) to approach natural microenvironmental features. Starting from real lung tissue, these features are captured in the TD-PCLS approach, thus providing a viable lung tissue platform, where most of the immune and structural cells are morphologically, spatially, and functionally/metabolically maintained over the observation period of several days, allowing us to follow tumor biology and treatment response in natural lung TME. Going beyond 3D models, TD-PCLS better represent the spatial complexity of TME by preserving features like blood vessels, extracellular matrix, and a diverse array of immune cells. In addition, TD-PCLS captures the genetic heterogeneity of tumors, a feature not adequately represented by conventional 3D models (11).

Despite the significant increase in the application of PCLS in lung physiology and pathology, along with advancements in culturing and preservation protocols (15, 28, 29), there have been relatively few studies using human TD-PCLS, especially those freshly derived from individual patients undergoing lung tumor resection (13). Dong et al. (30) employed a Krumdieck tissue slicer to cut 200 μm thick fresh lung tumor slices with a 5 mm diameter. They demonstrated the potential of tumor slices as a translational platform for nanoparticle delivery systems aimed at inhibiting telomerase activity in tumor cells. Specifically, they successfully delivered antisense 2-O-methyl-RNA carried by nanoparticles to approximately 50% of the cells within the tumor slices. Compared with our platform, Dong et al. did not culture the tumor slices for an extended period of time, initiating treatment only 24 hours after preparing the slices (30). Additionally, unlike our method, which utilizes low melting agar to fill the lung tissue and stabilize its architecture during the cutting process, they sliced the tumor tissues directly without agar filling, possibly provoking enhanced structural changes and stress responses. In our approach, we found the parenchymal structures to be conserved not only in the lung tumor areas, but also in the adjacent healthy parts of the lung. Despite these differences, similar to our findings, Dong et al. demonstrated the proliferative capacity of tumor cells within lung tissue/tumor slices (30). Although only freshly dissected TD-PCLS were used in our study to initially establish a viable and responsive ex vivo human lung tumor model system, new protocols for cryopreservation of tumor-derived PCLS, as reported in the context of idiopathic pulmonary fibrosis (31), offer promising opportunities to extend the utility of this model. Future work could explore cryopreservation of TD-PCLS as a means to create biobanks for personalized drug screening and retrospective mechanistic studies. Altogether, our study builds on this foundation by offering a more refined methodology that maintains tissue integrity and supports long-term culture, providing a more accurate model for studying lung cancer therapies.

More recently, Junk et al. employed an ex vivo tissue culture model to predict individual tumor responses to nivolumab, demonstrating that NSCLC tissue slices could maintain morphological characteristics and T cell function for up to 12 days (32). They utilized a semiautomated analysis to evaluate tissue responses to nivolumab, revealing alterations in Treg populations and tissue reorganization that correlated with nivolumab responsiveness (32). Similar to our findings, they observed patient heterogeneity in response to nivolumab, with some patients’ tumor slices not responding to the immunotherapy. Our method for producing TD-PCLS offers 2 main advantages over the approach used by Junk et al. Firstly, we preserve the parenchymal structure, including airway, vascular, and alveolar compartments, by using agar filling, which maintains the integrity of the lung architecture. Secondly, we do not use 3 mm punches, but work with larger tumor areas to reduce the impact of high intratumor variance in immunocomposition, which Junk et al. recognized as major handicap in their study (32). Including larger tumor areas is also essential for metabolic analysis. Additionally, by employing advanced multiplex imaging techniques, we mapped the spatial distribution and interactions of various immune cell types within the TD-PCLS. Overall, our findings not only align with Junk et al.’s observations of T cell function and response variability to nivolumab, but our methodological approach substantially enriches the explanatory power and informative value of the previous technique, providing detailed insights into cellular interactions within the lung TME.

In addition to biological heterogeneity, incorporating physiological readouts — such as tissue stiffness — into the TD-PCLS platform offers a promising strategy to enhance clinical relevance and translational value. It is well established that tumors and the TME are significantly stiffer than healthy tissue due to extracellular matrix remodeling and increased cellular contractility (33, 34). Recent advances, such as the biomechanical measurement approaches described by Kim et al. (35), have demonstrated how to quantify stiffness changes in PCLS model. Applying such approaches to TD-PCLS could not only enable longitudinal monitoring of mechanical properties during treatment ex vivo but also provide a platform for understanding tumor stiffness compared with healthy PCLS. The integration of mechanical profiling with cellular and molecular analysis of TME may offer a more comprehensive assessment of treatment response and could further support the use of TD-PCLS and TCS-PCLS in personalized therapy design.

Regarding the TCS-PCLS model, one of its significant advantages is the ability to cryopreserve healthy tumor PCLS for extended periods, ensuring that their viability and functional characteristics are maintained upon thawing, thus enabling consistent and reliable use in long-term cancer research studies. Previous studies have demonstrated that normal lung tissue can retain its structural integrity and cellular function after cryopreservation, making it a versatile resource for various experimental setup (36). Furthermore, healthy PCLS contain inherent immune and stromal cellular components that can interact with newly inoculated lung cancer cells, thus effectively simulating the natural TME (37). Our TCS-PCLS model enables detailed genetic and metabolic manipulation within this natural TME. This approach is not only valuable for understanding lung cancer pathobiology, but also offers major advantages for advancing precision medicine. By generating specific lung cancer cell lines based on the genetic profiles of individual tumors, we can introduce these cells into normal PCLS, creating a patient-specific model. Additionally, this system provides a platform for incorporating engineered immune cells, such as chimeric antigen receptor (CAR) T cells or reprogrammed macrophages (38), into the PCLS alongside cancer cells. This feature allows to study the direct interactions between engineered immune cells and cancer cells within the natural lung tissue context, providing further insights into the efficacy and mechanisms of immunotherapies. The ability to manipulate the immune components of the PCLS additionally enhances the model’s relevance for preclinical studies. Moreover, this TCS-PCLS model may overcome disadvantages due to limited availability of suitable TD-PCLS from patients. The observed migration of cancer cells in the TCS-PCLS model suggests that the agarose-filling approach used in our protocol does not inhibit cellular movement. The low melting agarose transitions from a gel to a fluid state at approximately 65°C. Therefore, at 37°C—the temperature used during ex vivo culture in an incubator—the agarose within the inflated lung remains in a gel state, preserving the structure of the alveoli and parenchyma (39). Additionally, the use of 1% low melting–point agarose likely plays a critical role in maintaining tissue integrity without creating a matrix that is too stiff to impede cellular migration. Higher concentrations of agarose (e.g., 2%–3%) have been reported to significantly increase stiffness and restrict cell movement (35), while our chosen concentration appears to strike an effective balance between structural support and biological accessibility. This is consistent with findings by Akram et al., who demonstrated epithelial cell migration during alveologenesis in PCLS using 1.5% low melting–point agarose — very similar to the concentration used in our study (40). Finally, we utilized both dispersed cancer cell lines and, in select experiments, preaggregated spheroids. The use of spheroids may enhance integration and viability due to improved cell-cell signaling and extracellular matrix (ECM) production. Future studies directly comparing single-cell seeding versus organoid or spheroid-based delivery will be valuable in determining whether the 3D structure contributes to more efficient engraftment and interaction within the PCLS microenvironment. By using a standardized source of healthy PCLS and introducing cancer cells/spheroids and immune components, we can create consistent and reproducible models for developing tailored, cancer cell–specific immunotherapies.

In conclusion, TD-PCLS emerge as a versatile and physiologically relevant ex vivo model that effectively recapitulates the complex lung TME. This model’s ability to sustain cellular viability, preserve immune constituents, and reflect the spatial and functional heterogeneity of lung tumors positions it as a powerful tool for both basic and translational lung cancer research. Furthermore, our TCS-PCLS model offers a sound alternative in scenarios where direct generation of TD-PCLS from patient samples may be possible.

Future studies could further refine this model by integrating patient-specific genetic backgrounds and exploring its applicability across different cancer types and therapeutic modalities. Ultimately, the adoption of TD-PCLS in preclinical research holds the potential to greatly improve the predictive accuracy of therapeutic responses, facilitating the development of more effective and personalized treatment strategies for lung cancer patients.

There are limitations to our study, including that, since the TCS-PCLS model utilizes lung tissue from cancer patients, premalignant changes may be present in the tissue, even at sites distant from the tumor. These changes could influence the baseline TME and associated cellular responses. To address this, we plan to conduct a future study comparing noncancerous tissues at varying distances from the tumor. This will help clarify how TME composition affects tumor-stroma and immune interactions within the model. One primary challenge in preparation of TD-PCLS is that not all human lung tumors are suitable for this method. Several factors including tumor type, size, patient consent, and pathologist assessment play critical roles in determining whether a lung tumor can be utilized for TD-PCLS preparation. Tumor size, in particular, determines the variability in the number of slices that can be harvested, with smaller tumors naturally yielding fewer or less viable samples. Consequently, the established methodological tools, such as seahorse, imaging, and FACS analysis, may not be applicable in each case. To mitigate this limitation, the use of TCS-PCLS model could be explored further, with healthy, fresh, or frozen PCLS being more readily available, though not fully mimicking the complexity and heterogeneity of the natural lung tumor architecture. However, TCS-PCLS does not fully represent the complexity of the architecture of the native lung tumor, in particular due to the lack of an intact vasculature (arteries, veins, and lymphatics) and the lack of dynamic recruitment of immune cells from the circulation. The inclusion of autologous PBMCs in a continuous artificial circulatory system could help to alleviate these limitations in future studies by partially restoring immune interactions. This should also be considered for the TD-PCLS model, where there is no circulation and no blood flow. Since no malignancy is induced in TCS-PCLS, the addition of cancer cells to healthy lung tissue corresponds to tumor progression or metastasis rather than tumorigenesis. To better model early stages of lung cancer, future studies should explore methods to induce malignancy in healthy lung tissue derived from lung cancer patients to provide a more physiologically relevant system for investigating tumor initiation.

Despite these challenges, the TD-PCLS platform represents a valuable tool for studying lung cancer under patient-specific natural microenvironmental conditions.

## Methods

### Sex as a biological variable

Sex was not included as a biological variable in the design or analysis of the data.

### TD-PCLS preparation and culture (Figure 1A)

The overall TD-PCLS procedure is summarized in Table 2 as the minimum reporting criteria table.

### TD-PCLS preparation

#### Preparing the agarose solution.

Dissolve 1% low melting–point agarose (A9414, Sigma) in DMEM/F12 medium (Ref: 21331020, Lot: 2436828, Gibco) (without additives) by boiling in a microwave. Transfer the dissolved agarose solution to 2 500 mL bottles. Place the tube in a beaker containing warm water, and then place the beaker in a 42°C water bath to maintain the agarose in a liquid state until use.

#### Agarose filling of the lung in the operation room.

The PCLS are prepared from surgically resected lung lobes from lung cancer patients (anatomical resections). Immediately after surgical removal, the flap is filled with 1% low-melting agarose through a bronchial opening using a flexible button cannula (B/BRAUN) until it is fully inflated. This is performed slowly using a 50 mL syringe. To prevent leakage of agarose, the button cannula is fixed with surgical clamps until the agarose has solidified (Figure 1B). Gradually apply ice onto the lungs to solidify the agarose. Remove the butterfly catheter and quickly close the hole with a needle holder. Place the agar-filled lung on ice and transfer it to the pathology lab for tumor evaluation.

#### Tumor evaluation with expert pathologist.

Cut the lung tissue based on established pathological evaluation protocols for lung tumors (Figure 1C). An in-house board-certified pathologist then examines the lung lobe to identify tumor, adjacent and non-tumor areas for both clinical and experimental purposes. Histologic samples are collected and the remaining tissue is transported to the laboratory for PCLS processing. Prior to sectioning, any visible agarose on the surface is carefully removed. The tissue is then cut into smaller blocks corresponding to tumor and adjacent healthy regions in DMEM/F12 media on ice for transferring before sectioning into slices by vibratome.

#### Cutting and culturing TD-PCLS.

Prepare the vibratome (Leica VT1200S): Clean and disinfect all vibratome components (blade holder, metal mount holder, tank, and surfaces) with 70% ethanol. Adjust the vibratome settings based on tissue quality: Thickness: 200 μm (if the tumor is hard to cut, try 400 μm and up to 500 μm). For healthy tissue, use maximum speed 1.5–2 mm/s; for tumor tissue, start at 0.8 mm/s and increase if necessary. Recommended amplitude is around 2.00. Use single mode to obtain slices.

Put enough ice on surrounding area of tank to cool the tank during the tissue cutting. Pour PBS (Ref: 14190-094, Lot: 2717604, Gibco) with 1% penicillin/streptomycin (P/S) (Ref: 15140-122, Lot: 192814, Gibco) + 0.1% amphotericin (Ref: 15290-018, Lot: 2313602, Gibco) into the vibratome tank where slices will be collected. Carefully place the razor blade and adjust its angle. The angle adjustment is dependent on the height of the tissue between 30–40 degree. Cut a block of approximately 8×8×8 mm from the normal/tumor tissue. Mount the tissue block on the metal holder of the vibratome using the glue (Best, KLEBSTOFFE). Keep the remaining tissue in the fridge or on ice (Figure 1D).

Prior to sectioning, any visible agarose on the lung surface is carefully removed. Start sectioning. If cutting quality deteriorates, change the blade (Wilkinson sword) or use another part of the blade. Transfer each slice immediately after cutting into a plate containing PCLS harvest medium (DMEM/F12 + 1% Pen/Strep + 0.1% Amphotericin). Place the plate on ice during sectioning.

After sectioning, transfer slices to a sterile plate with PCLS culture media (DMEM/F12 + 10% FBS (Ref: A5256801, Lot: 2575640H, Gibco) + 1% Pen/Strep + 0.1% Amphotericin) under laminar flow (Figure 1E). Incubate the slices in a tissue culture incubator (37°C, 5% CO_2_) for further analysis and experimental plans (Figure 1, E and F). Clean and disinfect all removable vibratome components. Wipe down nonremovable compartments with tissue paper.

### Metabolic flux analysis in TD-PCLS

We used the Seahorse XFe96 extracellular flux analyzer (Seahorse Bioscience, Agilent Technologies) to measure OCR. TD-PCLS was cut with 4 mm puncher (TED Pella, INC) and put in microplate spheroid XFe96 plate. Before the OCR measurement, TD-PCLS media was changed with XF RPMI media supplemented with glucose, pyruvate, and glutamine. Mitochondrial perturbation experiments were performed by sequentially adding 1.5 μM oligomycin, 2 μM FCCP (carbonyl cyanide 4-(trifluoromethoxy) phenylhydrazone), and 1 μM rotenone/antimycin. The OCR changes after addition of the substrate were calculated relative to the rate before injection. The OCR measurement compounds (103015-100) and ECAR analysis reagents (103020-100) were purchased from Agilent Technologies.

### FACS analysis of TD-PCLS

The single-cell suspensions were prepared from TD-PCLS using the Tumor Dissociation Kit (130-095-929, Miltenyi Biotec). For each condition, at least 2 TD-PCLS with 400 μm or 4 TD-PCLS with 250 μm should be selected. Accordingly, the enzyme mixture for each condition was prepared by adding 100 μL of enzyme H, 50 μL enzyme R and 15 μL of enzyme A in 4.7 ml of RPMI 1640 media (Ref: 21875-034, Lot: 2994571, Gibco). The enzyme mix was transferred into gentleMACS C Tube (130-093-237, Miltenyi Biotec). The TD-PCLS was chopped precisely into 2–4 mm small pieces then transferred into gentleMACS C Tube. The sample dissociation was started by placing the tubes on gentleMACS Dissociator (Miltenyi Biotec) by running gentleMACS program h_tumor_01, which should be stopped after 20 seconds then the dissociated samples were incubated for 30 minutes at 37°C under continuous shaker with 140 rpm. The dissociation procedure was stopped by adding 15 mL of RPMI 1640. Thereafter, the tube was centrifuged at 500*g* for 10 minutes at 4°C and the cell pellet was suspended in 20 mL RPMI 1640 media followed by filtering through 2 strainer filtering, first 70 μm and then 30 μm placed on a 50 mL tube. The cells were collected with centrifugation at 500*g* for 10 minutes at 4°C. Then, if necessary, red blood cells (RBC) were lysed by incubation with 1X RBC lysis buffer (Lot: 3199169, BD Pharma lyse) for 5 minutes followed by centrifugation at 500*g* for 10 minutes. The cell pellet was stained based on below plan and antibodies (Table 3).

Single-cell suspensions were prepared as mentioned above and then blocked with FcR blocking reagent (Miltenyi Biotec) in 0.5% PBS-BSA for 20 minutes, stained with fluorochrome-conjugated antibodies and analyzed on a FACSSymphony A5SE flow cytometer (BD Biosciences). Live single cells were identified by FSC/SSC characteristics. Data were analyzed using FlowJo V10 (TreeStar). All antibodies and secondary reagents were titrated to determine optimal concentrations. Comp-Beads (BD) were used for single-color compensation to create multicolor compensation matrices. For gating, fluorescence minus one control were used. The instrument calibration was controlled daily using Cytometer Setup and Tracking beads (BD Biosciences).

### Immunofluorescence staining and imaging

After culture, TD-PCLS were fixed with 4% Paraformaldehyde (PFA) in PBS for 10 minutes at RT, washed 3 times with PBS, and permeabilized with 0.5% Triton X-100 in PBS for 15 minutes. After again washing 3 times with PBS, TD-PCLS was blocked in 5% BSA for 1 hour, followed by incubation with primary antibodies as follows, CD68 (Abcam, #ab955), IL-8 (eBioscience, #14-7189-82), ALOX15 (Life technologies, #A2412), CD206, Cleaved Caspase 3 (Cell Signaling, #9664), Cytokeratin 18 (Abcam, #ab181597) and corresponding secondary Alexa Fluor-conjugated antibodies (Life technologies, #A11008, #A21422). Nuclei were counterstained with 4′,6-diamidino-2-phenylindole (Dapi). For Imaging, TD-PCLS were either mounted in Prolong Gold Antifade Mountant (Invitrogen) or transferred into a 8-well μ-Slide (ibidi) containing PBS. Imaging was performed with the SP8 MP Confocal Microscope (Leica), and images were analyzed by using the ImageJ software.

For assessment of proliferation, the Click-iT EdU Cell Proliferation Kit (Invitrogen) was used. For this, 20 μM EdU (5-ethynyl 2′-deoxyuridine) was added to the TD-PCLS and incubated for 6–10 hours prior fixation. Incorporated EdU was stained according to the manufacturers’ protocol followed by antibody staining and nuclear staining as described above.

### TCS-PCLS

A549 human cell line was obtained from the American Type Culture Collection (ATCC, Manassas) and maintained according to ATCC guidelines. A549-GFP spheroids were generated in “hanging drops.” For this, 80,000 cells were resuspended in 5 mL of culture medium with 20% Methycellulose (#M0262-100G, Sigma). Cell suspension was distributed as 25 μL drops on a petri dish lid and stored for 7–10 days upside down in a cell incubator to allow formation of 3D aggregates.

PCLS derived from the nontumor part of the lung was used as a mimic for human lung tumor development. Here, medium was removed from the PCLS and lung tumor cells (A549) that were stably transfected with green fluorescence protein (GFP; pCDH-CMV-MCS-EF1-copGFP-T2A-Puro Plasmid), were seeded on each slice (50,000 cells/30 μL or as spheroids, see above) (μ-Slide 8 Well high, iBidi) and allowed to settle down on the tissue. After 30 minutes, medium containing the desired treatment (here is conditional media harvested from naive M0, tumor-promoting M2 macrophages and anti-tumor M1 macrophages) was added and incubated at 37°C and 5% CO_2_ in a humid incubator. TCS-PCLS was used for time lapse imaging (24 hours) to assess the migratory behavior of the cancer cells, or was fixed with 2% Formaldehyde/0.2% Glutaraldehyde in PBS for 5 minutes at 4°C to preserve the GFP-staining, and imaged with the SP8 MP Confocal Microscope (Leica).

Time-lapse imaging was performed with the BZ-X810 fluorescence Microscope (Keyence) together with a Stage Top Incubator (Tokai Hit) for temperature, humidity and CO_2_ control. Analysis was done with ImageJ Software.

### Generation of human macrophages

Human macrophages were generated from PBMCs, as previously described (26, 38). Briefly, PBMCs were isolated from buffy coats obtained from the blood bank of the Universities of Giessen and Marburg Lung Center using Ficoll density gradient centrifugation. Platelets and RBC were removed by using RBC lysis buffer (BD Biosciences) and subsequently washing with phosphate buffered saline (PBS). The PBMCs were then seeded on 6-well plates (Sarstedt, Nümbrecht) in RPMI 1640 medium containing 1% P/S. After 2 hours, the nonadherent cells were removed and the remaining adherent cells were differentiated into macrophages over 10 days in macrophage complete medium RPMI 1640 medium containing 2% human serum and 1% penicillin/streptomycin. For macrophage polarization, antitumor M1 macrophages were generated by stimulating unpolarized naive M0 macrophages (M0) with LPS (100 ng/mL, Sigma) and interferon γ (IFN-γ) (100 U/mL, Roche) for 24 hours. Tumor-promoting M2 macrophages were generated by treating M0 macrophages with IL-4 (20ng/ml; VWR, Radnor) for 24 hours.

### LDH activity

LDH activity assays and staining of TD-PCLS were conducted as previously described (38, 41) The primary buffer consisted of 0.1 M Tris-maleate, pH 7.5, with 10% polyvinyl alcohol dissolved at 60°C with continuous stirring until the solution was fully clarified. The assay medium was freshly prepared with 0.45 mM methoxyphenazine methosulfate, 5 mM sodium azide, and 5 mM nitroblue tetrazolium chloride, each predissolved in a solution of 50% ethanol and 50% dimethylformamide and preheated to 60°C. For the LDH activity medium, substrates were added to the assay medium, specifically 150 mM sodium lactate and 3 mM NAD^+^. This activity medium was applied to cover the cryosectioned TD-PCLS. Enzyme reactions proceeded at room temperature until substantial staining was observed. Reactions were then halted by removing the incubation medium and washing the sections with PBS.

### Multiplex immunofluorescence and imaging on TD-PCLS

Multiplex immunofluorescence (mIF) staining was performed on slides containing 4-μm thick sections of TD-PCLS using antibodies conjugated with oligonucleotides and sequentially applied fluorescent reporters with the Phenocycler-Fusion system (Akoya Biosciences). Initially, formalin-fixed paraffin-embedded (FFPE) 4-μm TD-PCLS slides were subjected to deparaffinization and heat-induced antigen retrieval using a citric acid solution (pH 6.0). Autofluorescence was quenched utilizing a bleaching solution (30% H_2_O_2_) under 2 broad-spectrum LED light sources at 25,000 lux for 2 sessions of 45 minutes each. After that, the TD-PCLS slide was incubated overnight at 4°C with a cocktail of 16 antibodies conjugated with each unique oligonucleotides at optimized dilutions. Following manual staining, a flow cell was placed onto the slide, and the slide was secured onto a flow cell carrier, which was then inserted into the fusion stage of the Phenocycler-Fusion system. Sequential fluorescent reporters corresponding to the primary antibodies were prepared at optimized dilutions in a 96-well format, with each well containing 3 distinct reporters along with nuclear staining Dapi. Details of the biomarkers used in this study are provided in Table 4. A multicycle experiment was carried out using the phenocycler, a microfluidics device (Akoya Biosciences), which was fully automated and operated by software. Fluorescent reporters specific to the antibodies were deposited onto the TD-PCLS slide and imaged in spectrally distinct fluorescent channels using Phenocycler imager within the Phenocycler-Fusion system.

Image analysis was performed to distinguish various cell phenotype distributions within TD-PCLS. The procedure began with a quality assessment, involving a visual examination of the slide and a qualitative evaluation of each marker’s signal intensity and specificity relative to background noise. Nuclear segementation was performed using the StarDist deep learning technique with standard settings and the 2D_dsb2018 model on the Dapi-stained image. Following this, cytoplasmic segmentation was carried out using a 6-μm morphological expansion based on the nuclear mask. After cell segmentation, the mean intensity of each marker within individual cells was calculated using the segmentation masks and marker images. Protein expressions were normalized across all cells on a slide using z scoring, ensuring an average of 0 and a SD of 1 for each protein. Unsupervised clustering analysis was performed to identify cell clusters based on protein expression patterns using a GPU-accelerated Leiden algorithm. Identified cell clusters were manually annotated based on specific biomarkers expression on specific cell phenotype.

### Statistics

The statistical analysis was performed using Prism 9 software (GraphPad Software Inc.). A 2-tailed unpaired Student’s *t* test was used to compare 2 groups. Data are presented as mean ± SEM.

### Study approval

All lung tissues and blood samples utilized in this study were obtained with ethical approval from the Ethics Committee under file number AZ 58/15 of department of human medicine at Justus Liebig University Giessen in compliance with national regulations and the guidelines. In addition, a written declaration of consent was obtained from each patient (ethical reference number: AZ 58/15).

### Data availability

The data supporting the findings of this study are available in the Supporting Data Values file.

## Author contributions

RS and WS designed the study. RS supervised the project. SM, AK, SSP, AW, WS, and RS conceived and planned the experiments. CR, MAS, LS, DP, PD, LF, IA, SG, BE, AA, PK, HW, A Guenther, A Ghofrani, and FG provided lung cancer tissues. SM, AK, BA, RN, and AVC performed experiments. RS, WS, SM, and AK wrote the paper with input from all authors.

## Supplementary Material

Supplemental data

Supplemental video 1

Supporting data values

## Figures and Tables

**Table 1:**
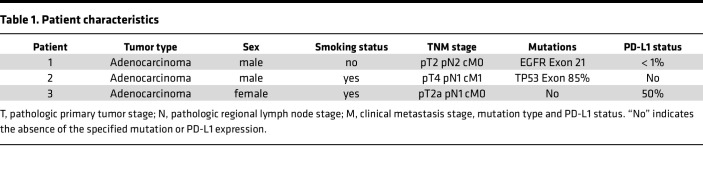
Patient characteristics

**Table 2:**
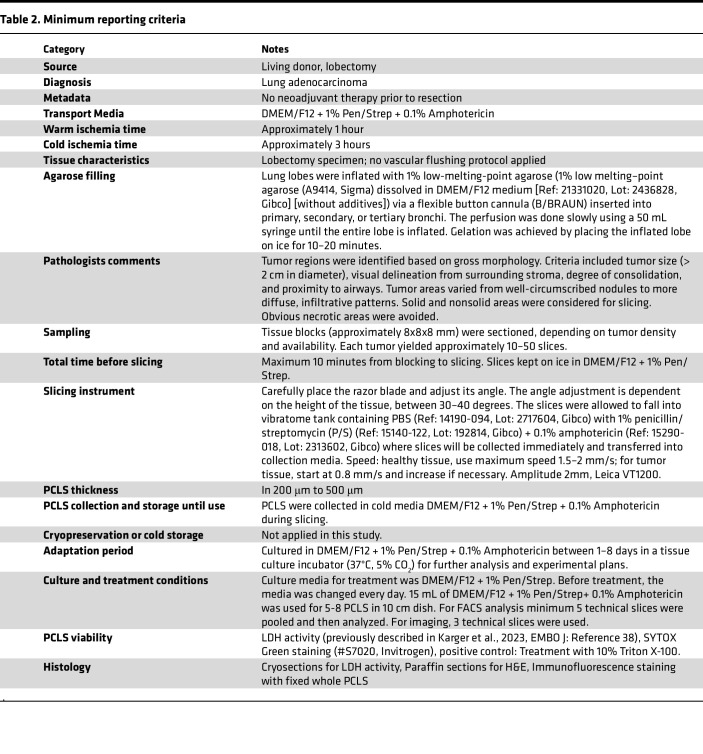
Minimum reporting criteria

**Table 3:**
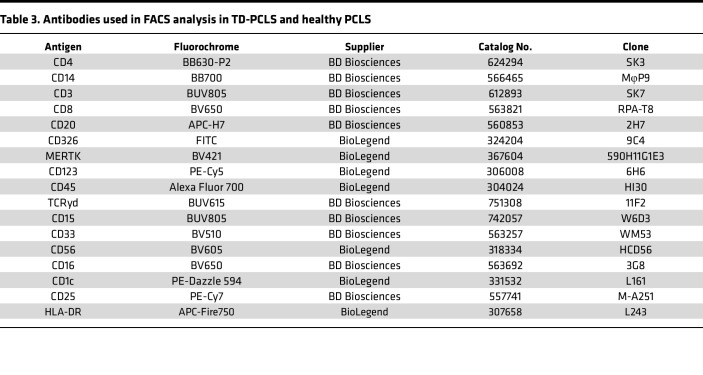
Antibodies used in FACS analysis in TD-PCLS and healthy PCLS

**Table 4:**
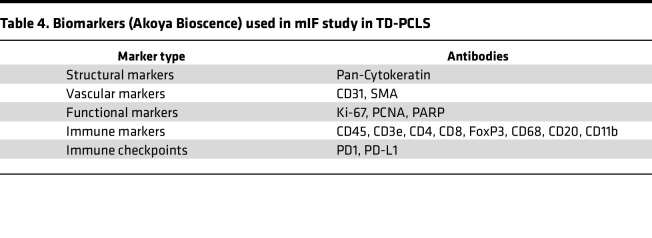
Biomarkers (Akoya Bioscence) used in mIF study in TD-PCLS

## Version 1 07/29/2025

In-Press Preview

## Version 2 09/09/2025

Electronic publication
