# Supplementary material for: Living human lung slices for ex vivo modelling of lung cancer
Source: JCI Insight. 2025 Jul 29;10(17):e190703. doi: 10.1172/jci.insight.190703 (PMC12487671; doi:10.1172/jci.insight.190703)
Supplement: Supplemental data [file jciinsight-10-190703-s151.pdf]

Supplementary Materials For

Living human lung slices for ex vivo modelling of lung cancer

Mansouri et al.
Corresponding author: Rajkumar Savai, rajkumar.savai@mpi-bn.mpg.de
Savai.rajkumar@innere.med.uni-giessen.de

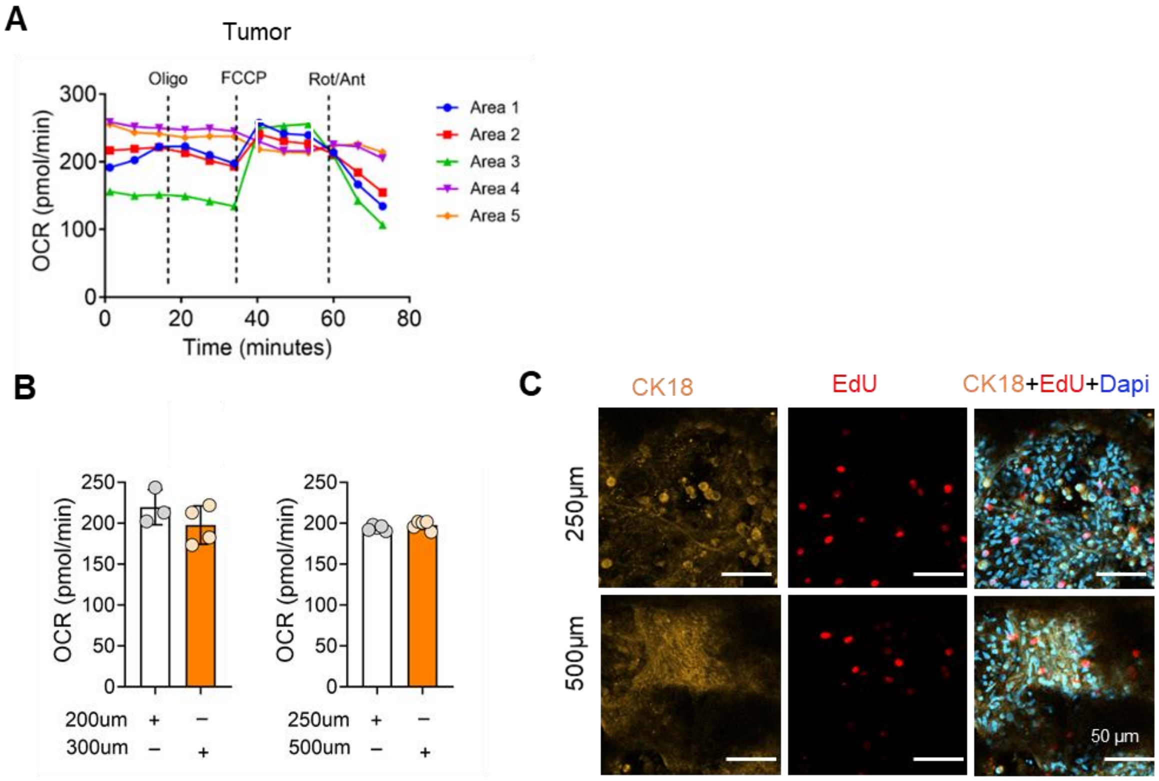

Supplemental Figure 1. TD-PCLS is a heterogeneous metabolic ecosystem. (**A**) Oxygen consumption rate (OCR) measured with Seahorse in various areas of tumor areas showing the metabolic heterogeneity of TD-PCLS (n=3 lung adenocarcinoma patients). (**B**) OCR of tumor regions from TD-PCLS which cut in 200um, 250um, 300um and 500um thickness (n=2 lung adenocarcinoma patients). Based on the tumor structure and solidity, one tumor were prepared in 250um and 300um and the second tumor was sliced in 250um and 500um then after two days OCR levels was measured by Seahorse analysis. (**C**) Tumor cell proliferation measured by EdU incorporation with subsequent staining in 250um and 500um sliced TD-PCLS. Cytokeratin 18 (CK18, tumor cell marker, orange), EdU (red), Dapi (blue). Scale bar = 50 µm.

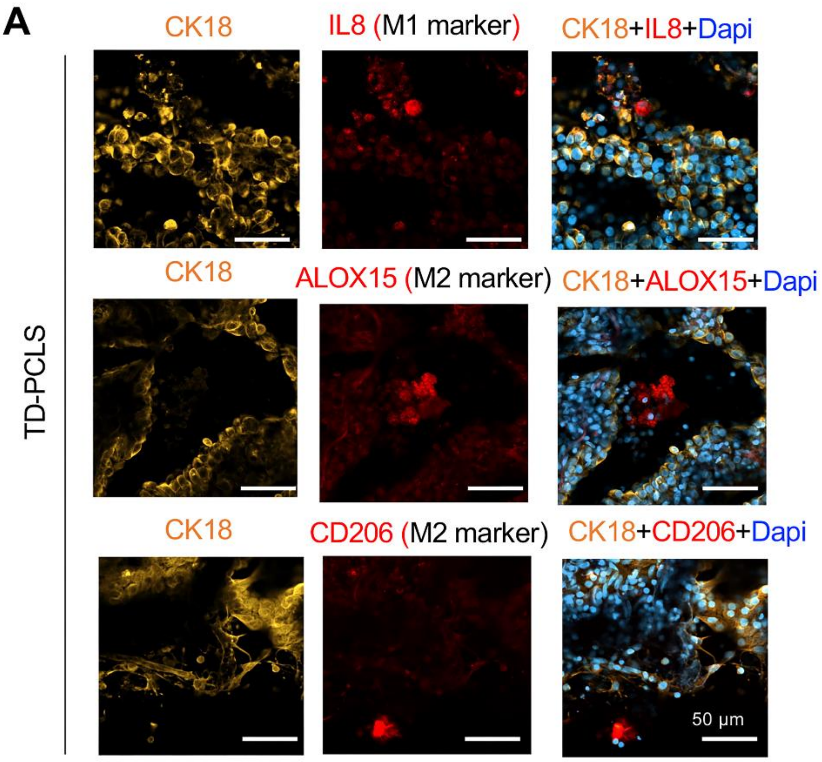

Supplemental Figure 2. Immunofluorescence analysis of macrophage subpopulations in TD-PCLS. (A) Immunofluorescence staining of cytokeratin 18 (CK18, tumor cell marker; orange) and macrophage subpopulation markers including IL8 (anti-tumor M1 macrophages marker, red), ALOX15 and CD206 (tumor-promoting M2 macrophages markers, red) in TD-PCLS. Dapi was used as nuclear dye (blue) (n=3 lung adenocarcinoma tumors). Scale bar = 50µm.

Supplemental Video 1. Time-lapse imagings of GFP-positive A549 cells on healthy PCLS. This video captures the dynamic movement of GFP-labeled A549 cells through the structural matrix of healthy PCLS over 24 hours. The footage illustrates the ability of tumor cells to infiltrate and migrate with the lung tissue of PCLS.
